# CGAS (Chloroplast Genome Analysis Suite): An automated python pipeline for comprehensive comparative chloroplast genomics

**DOI:** 10.1002/imo2.70093

**Published:** 2026-03-31

**Authors:** Abdullah, Rushan Yan, Xiaoxuan Tian

**Affiliations:** ^1^ State Key Laboratory of Chinese Medicine Modernization Tianjin University of Traditional Chinese Medicine Tianjin China; ^2^ Haihe Laboratory of Modern Chinese Medicine Tianjin China

## Abstract

Chloroplast Genome Analysis Suite (CGAS) is a comprehensive bioinformatics pipeline that streamline chloroplast genome analysis from raw sequencing reads to publication‐ready outputs. The suite integrates 14 specialized modules organized across three sequential phases. Phase 1 (Modules 1–4) handles genome assembly, quality control, annotation, gene normalization, and National Center for Biotechnology Information‐format conversion using tools such as fastp, GetOrganelle, PGA, and others. Phase 2 (Modules 5–13) enables batch comparative genomics, encompassing gene content comparison, genome structure characterization, codon usage analysis, amino acid composition, single‐nucleotide polymorphism detection, intron profiling, simple sequence repeat identification, and nucleotide diversity assessment—with R‐based visualizations integrated where graphical representation of results is required. Phase 3 (Module 14) performs robust phylogenetic inference via alignment and tree‐building programs, including MAFFT, MACSE, IQ‐TREE, and others. Together, CGAS transforms raw chloroplast genome data into comparative insights, providing researchers with an automated, reproducible, and scalable solution for comparative chloroplast genomics, available at https://github.com/abdullah30/Chloroplast-Genome-Analysis-Suite-CGAS.

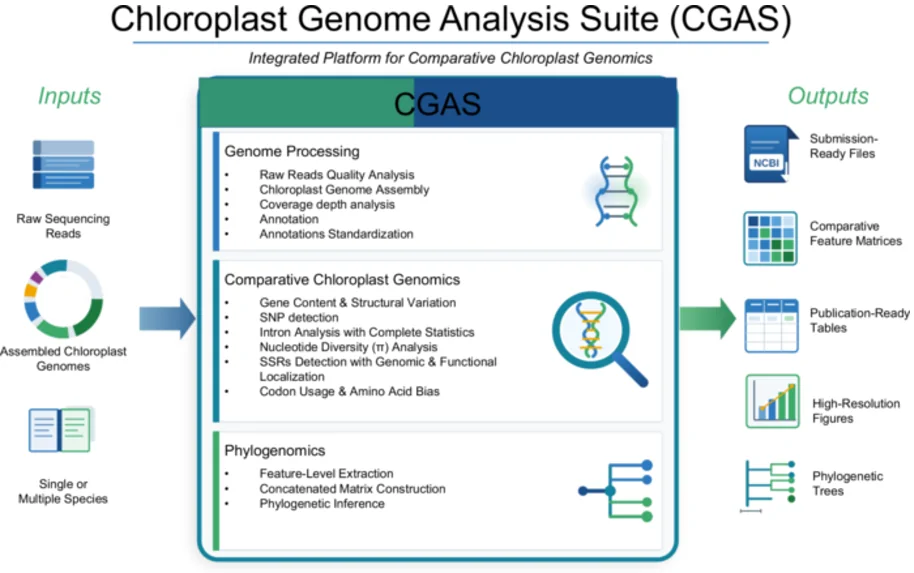

The chloroplast genome is a mostly conserved quadripartite molecule in which the large single copy (LSC) and small single copy (SSC) regions are separated by a pair of inverted repeats (IRs: IRb and IRa) [1, 2]. Owing to its moderate size, uniparental inheritance, and relatively stable gene content and organization, the chloroplast genome has become a cornerstone for studies of plant evolution, DNA barcoding, population genetics, phylogenetics, and phylogeography [1, 2, 3, 4]. Advances in high‐throughput sequencing technologies have transformed chloroplast genome sequencing from a specialized endeavor into a routine outcome of genomic projects, shifting the analytical bottleneck from genome generation to large‐scale comparative analysis.

Despite the availability of numerous tools for individual analytical tasks—such as raw‐read quality control, chloroplast genome assembly and annotation, coverage depth estimation, annotation validation, repeat detection, codon usage and amino acid analysis, nucleotide diversity estimation, and phylogenetic inference—comprehensive chloroplast genome studies still rely heavily on fragmented workflows. These workflows typically involve manual file conversions, tool‐specific post‐processing, and ad hoc scripting, often requiring manual figure generation in external tools. Such fragmentation reduces reproducibility, complicates batch analyses across multiple species, and increases the risk of biologically inconsistent or misleading results.

To address these challenges, we developed the Chloroplast Genome Analysis Suite (CGAS), a Python‐based pipeline designed as a unified, automation‐oriented backend for chloroplast genome assembly, annotation, annotation validation, preparation of submission files to the National Center for Biotechnology Information (NCBI), comparative chloroplast genomics, and phylogenetic analysis. Rather than proposing new algorithms, CGAS emphasizes the rigorous integration of established bioinformatics tools with newly developed Python scripts for various comparative analyses, with a strong focus on biological correctness, batch‐scale reproducibility, and generation of publication‐ready outputs.

Comparative chloroplast genome analyses commonly encompass raw‐read quality assessment, genome assembly, annotation, coverage evaluation, gene content comparison, genome structure characterization, codon usage and amino acid composition analysis, simple sequence repeat (SSR) detection, substitution profiling, intron characterization, nucleotide diversity estimation, and phylogenetic reconstruction [2, 5]. High‐quality standalone tools such as fastp [6] for read quality control, GetOrganelle for chloroplast genome assembly [7], and BWA and SAMtools [8, 9] for read mapping and coverage estimation have become widely adopted and are recognized for their robustness and analytical accuracy. In addition, Plastid Genome Annotator (PGA) enables batch annotation of chloroplast genomes from curated reference sets [10]. However, these tools are typically executed independently, requiring manual coordination of inputs, outputs, and intermediate files across multiple analytical stages.

At a higher level of abstraction, integrated platforms such as CPStools [5] and CPGView [11] have improved accessibility by providing streamlined interfaces for selected downstream analyses and visualization. CPStools offers convenient automation for several comparative tasks, while CPGView provides intuitive graphical summaries of chloroplast genome features. However, both platforms primarily focus on downstream analytical or visualization steps, and do not encompass the preparatory phase of chloroplast genome analysis, including raw‐read quality control, chloroplast genome assembly, coverage assessment, annotation validation, and preparation of submission‐ready files to NCBI. As a result, users rely on separate tools and manual coordination before data can be analyzed or deposited. In addition, CPStools lacks several analyses implemented in CGAS, including amino acid composition, substitution profiling, intron characterization, and comprehensive multi‐species gene content comparison. It also omits detailed genome structural summaries that incorporate GC content across the complete genome, LSC, SSC, and IRs, and functional gene categories. CPGView relies on individual file uploads and interactive processing. This approach constrains batch analyses and limits scalability for large comparative studies.

CGAS is a dedicated, end‐to‐end pipeline that integrates raw‐read processing, chloroplast genome assembly, annotation and annotation validation, preparation of NCBI‐compliant submission files, comparative chloroplast genomics, and phylogenetic inference, accepting standard chloroplast genome inputs (FASTQ, GenBank, or FASTA files) and producing complete, biologically informed outputs suitable for immediate dissemination. To our knowledge, it is among the first pipelines to address end‐to‐end chloroplast comparative genomics through 14 integrated modules. Here, we describe the design, implementation, and benchmark performance of CGAS, and demonstrate its utility for end‐to‐end chloroplast comparative genomics.

## 1 OVERVIEW OF CGAS

CGAS v1.0.1 implements 14 fully integrated modules, structured into a three‐phase workflow that spans chloroplast genome preparation (Modules 1–4), comparative genomics (Modules 5–13), and phylogenetic analyses (Module 14). Together, these modules cover the full spectrum of analyses routinely reported in comprehensive chloroplast comparative genomics studies, while ensuring methodological consistency, scalability, and reproducibility (Figure 1).

**Figure 1:**
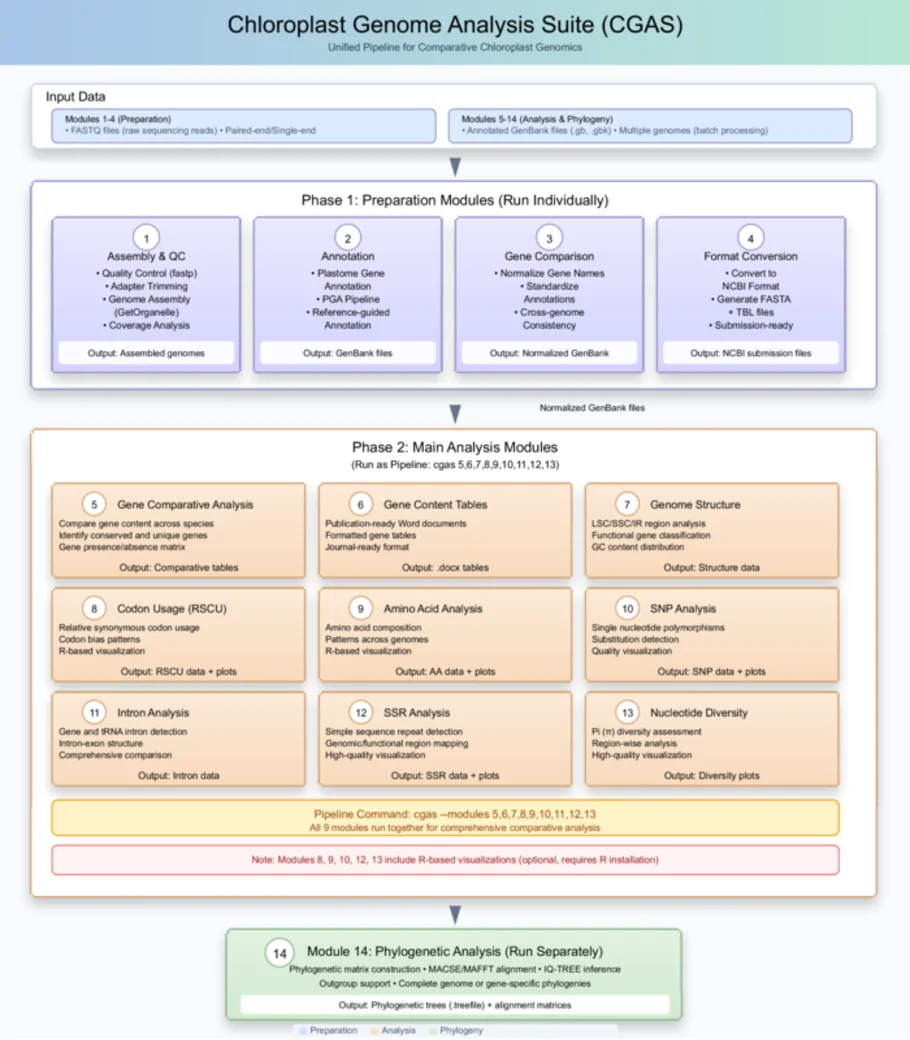
Workflow of CGAS (Chloroplast Genome Analysis Suite). The pipeline comprises 14 modules organized into three phases. Phase 1: Preparation modules (1–4) process raw FASTQ reads through assembly, annotation, gene normalization, and format conversion to generate standardized GenBank files; Phase 2: Main analysis modules (5–13) perform comprehensive comparative genomics including gene content analysis, genome architecture, codon usage, amino acid composition, substitution profiling, intron characterization, SSR detection, and nucleotide diversity assessment; Phase 3: Phylogenetic module (14) constructs phylogenetic matrices and infers evolutionary relationships. The pipeline ensures biological correctness through proper handling of trans‐spliced genes, IR duplications, and alternative gene names. All outputs are generated in publication‐ready formats (Excel, Word, R‐ready files) with structured directories.

## 2 PREPARATION WORKFLOW (MODULES 1–4)

Modules 1–4 constitute a dedicated preparation phase designed to standardize input data prior to downstream comparative analyses, with all steps executed in batch mode across multiple chloroplast genomes. Module 1 performs batch raw‐read quality control, chloroplast genome assembly, and coverage depth estimation through integrated execution of fastp, GetOrganelle, and BWA/SAMtools, with assembly completeness assessed automatically based on GetOrganelle's own output: assemblies are classified as complete if GetOrganelle produces a single circular contig or two contigs consistent with the SSC flip‐flop configuration (two orientational isomers of the small single‐copy region, a well‐recognized and expected output for chloroplast genomes). In all other cases—including assemblies yielding more than two contigs, non‐circular single contigs, or fragmented sequences—the assembly is flagged as incomplete, excluded from the output directory (07_assembled_genomes), and a warning is logged for each affected sample. Users encountering incomplete assemblies are directed to the GetOrganelle documentation for guidance on manual processing and troubleshooting. Coverage depth statistics (mean and median) are calculated genome‐wide using BWA and SAMtools and reported per sample; no minimum coverage threshold is imposed by default, and users are advised to inspect the per‐sample coverage reports and apply study‐specific thresholds before proceeding to downstream analyses. To facilitate manual inspection, Module 1 retains the sorted, indexed BAM file (04_mapping/{sample}/{sample}.sorted.bam and its.bai index) for each sample, which can be loaded directly into standard genome browsers such as Integrative Genomics Viewer (IGV) or Tablet for visual verification of coverage uniformity across the chloroplast genome. Additionally, the extracted chloroplast reads are saved as compressed FASTQ files (05_cp_reads/{sample}/), providing a lightweight dataset that can be rapidly re‐mapped to any reference using external tools for independent coverage verification; because only chloroplast‐derived reads are retained rather than the full sequencing library, this re‐mapping is computationally trivial and can typically be completed within minutes. Only assemblies passing the completeness criteria are retained for subsequent analyses. Module 2 annotates assembled genomes in batch using PGA with curated reference datasets, producing biologically consistent annotations of protein‐coding genes, transfer RNAs, and ribosomal RNAs. Module 3 normalizes gene annotations across all genomes by resolving alternative gene names, identifying missing or extra features, and explicitly accounting for intron presence or absence, thereby harmonizing annotations from de novo assemblies and genomes retrieved from NCBI. This normalization step ensures comparability of gene models across datasets and prevents downstream analytical bias arising from annotation inconsistencies. Normalization outputs also provide users with quality diagnostics to guide manual curation and distinguish genuine gene or intron loss from annotation errors. Module 4 performs final annotation validation in batch and converts curated GenBank files into NCBI‐compliant FASTA and TBL formats, facilitating large‐scale submission while minimizing annotation‐ and formatting‐related errors.

## 3 COMPARATIVE GENOMICS ANALYSES (MODULES 5–13)

Modules 5–13 perform core comparative analyses on curated GenBank files. Module 5 summarizes gene content across species, distinguishing functional genes from pseudogenes and accounting for IR‐mediated duplications without inflating counts. Module 6 produces formatted gene content tables suitable for direct inclusion in manuscripts. Module 7 characterizes chloroplast genome architecture by identifying LSC, SSC, and IR regions, calculating their lengths, and determining GC content at both regional and functional levels, including protein‐coding genes, tRNAs, and rRNAs.

Modules 8–13 focus on sequence composition and variation. Module 8 analyzes relative synonymous codon usage (RSCU values calculated from all annotated coding regions of protein‐coding genes of each chloroplast genome collectively, at their full length; raw codon counts per species are provided in individual per‐species output files for independent verification), Module 9 assesses amino acid composition (frequencies derived from translation of all coding regions of protein‐coding genes per genome); for both modules, gene‐length normalization was intentionally omitted to preserve biologically meaningful variation attributable to differences in gene content, IR boundary shifts, gene deletions, and pseudogenization events across taxa, Module 10 profiles substitutions (transitions, transversions, and Ts/Tv ratios via strictly pairwise sequence comparisons), Module 11 examines intron structure in protein‐coding genes and tRNAs, Module 12 detects SSRs with classification by motif type, genomic location, and functional context, and Module 13 estimates nucleotide diversity (π) across coding and non‐coding regions using MAFFT v7 [12]. All modules generate publication‐quality tables and figures; all statistical computations and tabular outputs are performed by the Python modules, which also export structured data files (CSV and TXT) that are consumed by automatically generated R scripts solely for producing the publication‐quality figures. The R scripts are additionally provided for optional manual customization of visualizations.

## 4 PHYLOGENETIC ANALYSIS (MODULE 14)

Phylogenetic analysis (Module 14) extends CGAS beyond descriptive comparative genomics by constructing phylogenetic matrices using feature‐level extraction. Each protein‐coding gene, intron, and intergenic spacer (IGS) is extracted and aligned individually, and the resulting alignments are concatenated to form the final matrices. Sequence alignment is performed using MAFFT for all regions by default, while an optional codon‐aware alignment using MACSE is available for protein‐coding genes to preserve reading frame integrity and minimize frame‐shift artifacts. The concatenated matrices are used for maximum‐likelihood phylogenetic inference with IQ‐TREE, with user‐defined outgroup specification. Substitution models are selected automatically per partition using ModelFinder Plus (–m MFP), and branch support is assessed with 1000 ultrafast bootstrap (UFBoot) replicates (–bb 1000) and 1000 SH‐aLRT replicates (–alrt 1000). UFBoot trees are additionally optimized by nearest‐neighbor interchange (–bnni) to reduce the impact of model violations. Final alignment matrices and inferred phylogenetic trees are saved in standard formats (FASTA and Newick, respectively) for immediate downstream use.

## 5 OUTPUT STRUCTURE AND PERFORMANCE

Each module generates results in a dedicated, systematically named output directory, maintaining strict separation between input genomes and analytical outputs. All results are produced automatically in structured formats, including Excel spreadsheets, Word documents, aligned sequence files, and high‐resolution figures. For most analytical modules, species‐specific raw outputs are generated alongside combined summary files, allowing transparent cross‐checking of intermediate and final results.

Benchmark testing demonstrated that CGAS Modules 3–13 processed 10 chloroplast genomes (~150 kb each) in under 10 min on a Dell Inspiron 15 3530 laptop equipped with an Intel Core i5‐1335U processor (13th generation, 10 cores with 12 threads, up to 4.6 GHz boost frequency) and 32 GB (DDR4) RAM. Complete analyses of 50 genomes were completed in approximately 50 min, with nucleotide diversity estimation representing the most time‐consuming step due to gene extraction, multiple sequence alignment, and nucleotide diversity calculations. For Module 1 (chloroplast genome assembly with GetOrganelle, raw read quality analysis with fastp, and coverage depth calculation using BWA and SAMtools) and Module 2 (annotation integration via PGA), CGAS does not reduce the intrinsic computational time of the underlying tools. Instead, it automates their sequential execution and supports batch processing, eliminating the need for users to manually run repetitive commands for each genome, hence, in practice, saving considerable analysis time between analytical steps. Similarly, Module 14 (phylogenetic analysis) streamlines the extraction of sequences and construction of phylogenetic matrices, while alignment with MAFFT or MACSE [13] and tree inference still require the standard runtime of these tools. Across all modules, CGAS applies consistent processing logic and parameter settings, ensuring reproducible and biologically coherent results across datasets.

CGAS is not intended to replace specialized visualization, annotation, and analysis platforms or tools such as CPStools [5], CPGView [11], Chloroplot [14], OrganellarGenomeDRAW [15], PGA [10], or GeSeq [16]. Rather, it provides a methodological backbone for comparative chloroplast genomics by integrating analyses that are otherwise scattered across multiple standalone tools or performed manually, unifying them into a single, reproducible workflow. CGAS supports end‐to‐end analyses—from raw read quality assessment and genome assembly through annotation, comparative genomics, and phylogenetic inference—while emphasizing batch processing, biological consistency, and analytical coherence. In this way, CGAS complements existing resources rather than duplicating their functionality.

A key strength of CGAS lies in its explicit handling of biological edge cases that are often overlooked or inconsistently treated in ad hoc workflows, including IR‐mediated gene duplication, trans‐spliced genes (e.g., *rps12*), and annotation inconsistencies among publicly available chloroplast genomes. By embedding biologically informed logic directly into each analytical module, CGAS minimizes the risk of misleading summaries, particularly in comparative studies involving large numbers of species. Its modular architecture enables flexible yet standardized analyses: upstream preparation modules (assembly, annotation, normalization, and validation) are clearly separated from downstream comparative and phylogenetic modules, allowing users to curate high‐quality input data while preserving reproducibility. All statistical computations—including RSCU values, amino acid frequencies, substitution counts, SSR tallies, and nucleotide diversity estimates—are performed entirely by the Python modules and saved as structured CSV and TXT files. These files are then read by automatically generated R scripts, whose sole function is to produce the publication‐quality figures; R performs no independent statistical analysis. The R scripts are additionally provided for optional manual customization of visualizations, reducing reliance on external plotting workflows.

A practical consideration for Module 1 is that assembly completeness is determined directly from GetOrganelle's own output—based on contig circularity and number—rather than by imposing independent size‐based thresholds. Similarly, no universal minimum coverage threshold is enforced by CGAS, because appropriate coverage requirements vary with sequencing technology, library complexity, and the intended downstream analyses. Users are therefore advised to examine the per‐sample coverage statistics reported by Module 1 and apply study‐specific thresholds prior to downstream analysis. To support this, Module 1 retains the sorted, indexed BAM file for each sample, enabling direct visualization of coverage uniformity in tools such as IGV or Tablet, and also saves the extracted chloroplast reads as lightweight FASTQ files that can be independently re‐mapped within minutes using any preferred tool. This design keeps the pipeline broadly applicable while ensuring that coverage data are always transparently reported and visually verifiable, supporting informed decisions during data curation.

CGAS represents a substantial advance in chloroplast genome analysis by integrating 14 essential modules—spanning assembly, annotation, validation, comparative genomics, and phylogenetic inference—into a single, automation‐driven framework, thereby simplifying complex workflows while preserving biological accuracy and reproducibility. As chloroplast genome datasets continue to expand in size and taxonomic breadth, unified analytical frameworks such as CGAS will become increasingly important for maintaining methodological rigor across evolutionary, taxonomic, and applied plant genomics research. CGAS is freely available, extensible, and actively maintained, making it particularly well‐suited for large comparative studies, meta‐analyses, and projects where transparency and reproducibility are essential.

## AUTHOR CONTRIBUTIONS

**Abdullah**: Conceptualization; software development; data curation; validation; writing—original draft; formal analysis. **Rushan Yan**: Testing; validation; data curation. **Xiaoxuan Tian**: Conceptualization; writing—review and editing. All authors have read the final manuscript and approved it for publication.

## CONFLICT OF INTEREST STATEMENT

The authors declare no conflicts of interest.

## AI STATEMENT

AI‐assisted tools, including ChatGPT (v5), Claude (Sonnet v4.5), and DeepSeek (v3.2), were used during script development, debugging, and language refinement of the manuscript. All AI‐assisted outputs were carefully reviewed, validated, and edited by the authors to ensure technical accuracy and scientific rigor. The authors take full responsibility for the content of this work.

## ETHICS STATEMENT

No animals or humans were involved in this study.

## Supporting information

Supporting Information Method.

## Data Availability

CGAS is released under the MIT license, and the full source code is freely available at https://github.com/abdullah30/Chloroplast-Genome-Analysis-Suite-CGAS. Comprehensive documentation, including installation instructions, module‐specific usage guides, and example workflows, is provided in the repository. In addition, the input and output files are provided in Figshare at https://doi.org/10.6084/m9.figshare.31267456. The tutorial of CGAS is available at https://www.bilibili.com/video/BV18LcazxEir/ (Bilibili) and https://www.youtube.com/watch?v=2Q4B7uCGkQE (YouTube). Supplementary Method (methods, graphical abstract, slides, videos, Chinese translated version, and updated materials) may be found in the online DOI or iMetaOmics Science http://www.imeta.science/imetaomics.
