# Supplementary material for: CGAS (Chloroplast Genome Analysis Suite): An automated python pipeline for comprehensive comparative chloroplast genomics
Source: IMetaOmics. 2026 Mar 31;3(2):e70093. doi: 10.1002/imo2.70093 (PMC13674443; doi:10.1002/imo2.70093)
Supplement: Supplementary file 1 [file IMO2-3-e70093-s001.docx]

Supporting Information to

**CGAS (Chloroplast Genome Analysis Suite): An Automated Python Pipeline for Comprehensive Comparative Chloroplast Genomics**

**Running title**: CGAS: Chloroplast Genome Analysis Suite

Abdullah^1,2*^, Rushan Yan^1,2^, Xiaoxuan Tian^1,2*^

^1^State Key Laboratory of Chinese Medicine Modernization, Tianjin University of Traditional Chinese Medicine, Tianjin 301617, China

^2^Haihe Laboratory of Modern Chinese Medicine, Tianjin 301617, China

*****Correspondence: [abdullah@tjutcm.edu.cn](mailto:abdullah@tjutcm.edu.cn), [abd.ullah30@yahoo.com](mailto:abd.ullah30@yahoo.com) (Abdullah), [tian_xiaoxuan@tjutcm.edu.cn](mailto:tian_xiaoxuan@tjutcm.edu.cn) (Xiaoxuan Tian)

**METHODS**

**Implementation and requirements**

CGAS is a modular, command-line-driven pipeline designed for large-scale chloroplast genome analyses. Implemented in Python, it integrates established bioinformatics tools for genome assembly, alignment, annotation, and phylogenetic inference. Depending on the analysis stage, CGAS operates either on raw sequencing reads (Module 1) or on curated GenBank and FASTA files (Modules 2–14). Core Python functionality relies on Biopython for sequence parsing and feature handling, NumPy and pandas for numerical computation and data manipulation, OpenPyXL for spreadsheet generation, and python-docx for producing publication-ready Word tables. Specialized computational tasks—including read preprocessing, assembly, alignment, and phylogenetic reconstruction—are delegated to external tools.

Multiple external bioinformatics tools are integrated into the CGAS workflow and executed automatically rather than manually. Raw-read quality control, chloroplast genome assembly, and coverage depth estimation (Module 1) are performed through coordinated execution of fastp, GetOrganelle, BWA, and SAMtools. Chloroplast genome annotation (Module 2) requires PGA. Multiple sequence alignments are generated using MAFFT (Modules 13 and 14), with optional codon-aware alignment using MACSE for phylogenetic analyses (Module 14). Maximum-likelihood phylogenetic inference is conducted with IQ-TREE. For visualization-oriented modules—including codon usage, amino acid composition, substitution patterns, simple sequence repeats, and nucleotide diversity—CGAS automatically generates and executes R scripts to produce consistent, publication-quality figures, while also providing R scripts for optional manual curation and customization.

AI-assisted tools, including ChatGPT (v5), Claude (Sonnet v4.5), and DeepSeek (v3.2), were used during script development, debugging, and manuscript language refinement. All AI-assisted outputs were manually reviewed, validated, and, where necessary, modified by the authors to ensure technical correctness and scientific rigor.

**Input requirements**

CGAS accepts standard chloroplast genome data formats and is optimized for directory-based batch processing. Module 1 requires paired-end or single-end FASTQ files as input. Module 2 operates on FASTA files generated by Module 1 or on FASTA files from previously published genomes requiring reannotation. Modules 3–14 primarily operate on annotated GenBank files (extensions .gb, .gbk, or .genbank) containing standard feature annotations, with the exception of Module 10 (substitution profiling), which requires pairwise-aligned FASTA files (each containing exactly two sequences) as input. Where necessary, CGAS automatically generates aligned FASTA files for downstream analyses, eliminating the need for manual alignment preparation. All modules are designed to process multiple genomes simultaneously by operating on directories rather than individual files, enabling scalable comparative analyses.

**Biological correctness and annotation handling**

Biological correctness is a core design principle of CGAS. The pipeline explicitly handles trans-spliced genes—most notably *rps12*—by recognizing split annotations and reporting them without artificial duplication. IR-mediated gene duplication is appropriately accounted for during gene content summarization, preventing inflation of gene counts. CGAS further distinguishes functional genes from pseudogenes using GenBank annotation qualifiers and incorporates safeguards to identify and exclude spurious or inconsistent annotations. Collectively, these measures ensure that comparative summaries reflect genuine biological variation rather than annotation artifacts.

**Availability and requirements**

Project name: Chloroplast Genome Analysis Suite (CGAS)

Project home page: https://github.com/abdullah30/Chloroplast-Genome-Analysis-Suite-CGAS

Operating system: Platform independent (Linux, macOS, Windows/WSL2)

Programming language: Python ≥ 3.9

Key dependencies: Biopython (≥ 1.79), pandas (≥ 2.0), NumPy (≥ 1.24), OpenPyXL (≥ 3.1), python-docx (≥ 0.8.11)

External tools: fastp (>= 0.23.0), GetOrganelle (>= 1.7.0), BWA (>= 0.7.17), SAMtools (>= 1.15), BLAST >= 2.12.0, PGA, MAFFT (>= 7.490), MACSE (optional), IQ-TREE (>= 2.2.0), openjdk >= 11 (required for MACSE); R programming (>= 4.0.0)

License: MIT
